# Facilitators and barriers in the rehabilitation process described by persons with spinal cord injury: a deductive-inductive analysis from the Finnish spinal cord injury study

**DOI:** 10.1080/07853890.2024.2303398

**Published:** 2024-01-17

**Authors:** Susanna Tallqvist, Kaarina Eskola, Anni Täckman, Anna-Maija Kauppila, Eerika Koskinen, Heidi Anttila, Marketta Rajavaara, Jari Arokoski, Sinikka Hiekkala

**Affiliations:** a Doctoral Programme in Population Health, University of Helsinki, Helsinki, Finland; b Doctoral Programme in Social Sciences, University of Helsinki, Helsinki, Finland; c Department of Rehabilitation and Psychosocial Support, Tampere University Hospital, Tampere, Finland; d The Finnish Association of Spinal Cord Injured Akson, Helsinki, Finland; e Department of Medical Rehabilitation/Spinal Cord Injury Outpatient Clinic, Oulu University Hospital, Oulu, Finland; f Department of Neurosciences, Tampere University Hospital, Tampere, Finland; g Public Health and Welfare Department, Knowledge Management and Co-creation Unit, Finnish Institute for Health and Welfare (THL), Helsinki, Finland; h Faculty of Social Sciences, University of Helsinki, Helsinki, Finland; i Department of Internal Medicine and Rehabilitation/Spinal Cord Injury Outpatient Clinic, Helsinki University Hospital, Helsinki, Finland; j Faculty of Medicine, University of Helsinki, Helsinki, Finland; k The Finnish Association of People with Physical Disabilities, Helsinki, Finland; l Faculty of Sports and Health Sciences, University of Jyväskylä, Jyväskylä, Finland

**Keywords:** Spinal cord injury, rehabilitation, facilitator, barrier, communication, interaction, qualitative content analysis

## Abstract

**Background:**

This study aimed to determine the factors that promote and facilitate rehabilitation as well as challenges and possible barriers perceived by people with spinal cord injury (SCI).

**Materials and Methods:**

This study was part of a larger, mixed-method Finnish Spinal Cord Injury (FinSCI) study. We interviewed 45 persons with SCI representing participants from the FinSCI study and used a qualitative approach and a deductive-inductive content analysis to analyse the data.

**Results:**

We identified 28 facilitators and 19 barriers in the rehabilitation process. The majority of the facilitators and barriers were related to the rehabilitation planning phase. There were more barriers than facilitators in applying for and accessing treatment or rehabilitation and during the implementation of rehabilitation. Facilitators consisted of successful, realized, or planned treatments or rehabilitation events; clear goals; multidisciplinary teamwork; support and monitoring in various changing situations; and the rehabilitees’ own capabilities and activities, among other things. The barriers included delays, challenges and deficiencies in the planning and implementation of treatment or rehabilitation; the lack of different skills; and resources of rehabilitation professionals; and different personal factors, which made the rehabilitation process cumbersome.

**Conclusions:**

Good communication and interaction between stakeholders are crucial for the progress of rehabilitation.

## Introduction

A spinal cord injury (SCI) can be traumatic (TSCI), resulting from an accident or injury, or nontraumatic (NTSCI), resulting from a congenital disorder, disease, or degenerative condition [1]. The care and rehabilitation of a person with SCI starts with rescue or the diagnosis of a disease, followed by acute treatment, rehabilitation, and continued life-long care with monitoring visits and independent exercise. Inpatient rehabilitation periods have an important role in improving functional status and optimizing independence, as well as helping persons with SCI return to their communities [2]. In Finland, there are three university hospitals, Helsinki (HUS), Tampere (Tays), and Oulu (OYS), that are responsible for acute phase care and immediate rehabilitation for people with SCI. Their actions are based on the Health Care Act and government decree [3]. The length of rehabilitation during acute and subacute stage (immediate rehabilitation) depends on several issues, like the level and severity of SCI and can vary from a few days to months. In addition to nurses and doctors, many different professionals, such as physiotherapist, occupational therapist, speech therapist, social worker, and psychologist participate in patient care and rehabilitation. These three spinal units are also responsible for lifelong multidisciplinary treatment and monitoring. Units provide specialized care for SCI-related health problems, and counsel and plan the further goals, both for inpatient and for outpatient rehabilitation, together with all stakeholders. Outpatient rehabilitation includes mostly physical or occupational therapy and mainly takes place at the service provider’s premises or at the rehabilitee’s home.

Based on previous publications of the Finnish Spinal Cord Injury Study (FinSCI), it is known that persons with SCI have more comorbidities than the rest of the Finnish population and they suffer from several secondary health conditions [4] and pain [5] due to their SCI. Employment rate among SCI population in Finland is low (26.5%) [6]. The aim of FinSCI was to help to develop the care and rehabilitation policies for the SCI population by gaining understanding of the lived experience of people with SCI [7]. In recent years, there has been increasing interest among researchers to study the barriers and facilitators in the lives of persons with SCI. Scientific publications with qualitative research samples have been published, e.g. related to bladder [8] and bowel [9] functions, pain [10], work and employment [11–13], education [14], and social participation [15–17]. However, there are very few qualitative publications from SCI rehabilitees’ perspectives in which facilitators and barriers are described during the whole rehabilitation process, meaning from accessing rehabilitation to independent exercising at the last phase of the process. Mlenzena et al. [18] published a systematic review in 2013 about barriers to and facilitators of rehabilitation services and found two studies [19,20] in which people with SCI, among other persons with neurological conditions, were involved. A quantitative study by Zongjie et al. [20] identified facilitators, such as the provision of information, doctors with good skills, easy access to doctors, good understanding, confidence in the value of rehabilitation services, and easily accessible rehabilitation services. In a qualitative study, Kroll et al. [19] identified structural-environmental barriers, such as facilities, equipment, procedural accessibility issues, and transportation and process barriers, such as problems with scheduling appointments and patient-provider communication, the lack of a professional manner, disability-specific knowledge and personal motivation among other things that prevented the use of health care services. Thus, more research is needed from the point of view of rehabilitees to increase person-centeredness in rehabilitation, to improve rehabilitee experience, and to support and develop the success of the rehabilitation process and services.

Unforeseen factors, motivation related to the rehabilitee’s life situation, interactive cooperation between the rehabilitation professionals and responsible administrative sectors play an important role in rehabilitation process [21]. Earlier research from Finland [22] and international studies [23] have shown that persons who need many health care services suffer from a fragmented system and the resulting lack of continuity in care and services. The aim of this study, as part of the FinSCI, was to identify factors related to the rehabilitation process and describe the factors that promoted and facilitated rehabilitation as well as challenges and possible barriers perceived by people with SCI.

## Materials and methods

The FinSCI study was a mixed-method collaborative study which detailed protocol, precise patient selection process, ethical considerations, and other parts of the mixed-method study have been presented in a separate publication [7]. The FinSCI study was conducted by The Finnish Association of People with Physical Disabilities, The Finnish Association of Spinal Cord Injured Akson, The Finnish Institute for Health and Welfare (THL), and the SCI outpatient clinics at three university hospitals (Oulu, Tampere, and Helsinki). The purpose of the FinSCI study was to identify the factors that are related to the health and functioning of persons with SCI, environmental factors causing challenges regarding accessibility, and how these factors are interconnected [7].

### Theoretical framework for the analysis

**Figure 1.**
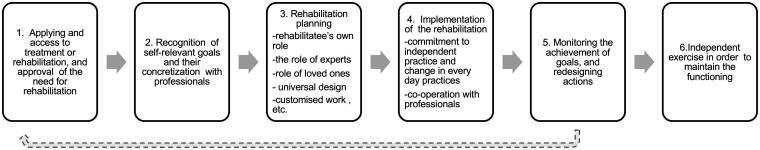
Rehabilitation process. The process was modified from the original figure from Autti-Rämö, 2021 [21].

This study had a qualitative design. The data collected from semistructured interviews were analysed by using a qualitative content analysis with a deductive-inductive approach as described by Elo and Kyngäs [24]. The rehabilitation process described by Autti-Rämö [21] was utilized to organize the results into six different phases, like presented in the Figure 1. This process has many similarities with the well-known healthcare management cycle of Wade [25], which is added as the last part of the process an independent exercise to maintain functioning. In this process rehabilitation and services are provided with the person [21], and it has also a lot in common with person-centred rehabilitation model, which is recommended for guiding rehabilitation services delivery and organization [26].

In this study, rehabilitation is understood as a process that progresses with a purpose and includes acute, subacute, and further rehabilitation. The process starts with the need for rehabilitation identified by a professional, and a part of the process includes setting a goal and rehabilitative measures to achieve the goal. The rehabilitee and experts plan the rehabilitation process together. The aforementioned factors must be considered for the process to be implemented in a timely and smooth manner (Figure 1) [21]. The process can end at the last phase, which is an independent exercise to maintain a person’s functioning, or it can start from the beginning in the fifth phase if, as a result of monitoring achievement toward the goal, it is found that the need for rehabilitation should be reassessed, as shown by the arrow pointing right in Figure 1.

### Participants

The participants (45 persons) were chosen among the respondents from the FinSCI study. The eligible population from the FinSCI study included 1,772 people and the final number of participants was 884, the inclusion criteria is presented elsewhere [7]. A purposeful maximum variation sampling was used, and 15 people were selected from each hospital district (Helsinki, Tampere, and Oulu), who would represent the area as truthfully and versatile as possible, based on the register data collected at the start of FinSCI [7]. ST selected participants based on the predetermined criteria; The primary criteria were time since injury and age. The aim was to select 5 participants for each injury time period group (1-5 years since the SCI, 6–10 years since the SCI, > 11 years since the SCI) and three participants for each age group (20–30 years, 31–45 years, 46–60 years, 61–75 years, and > 75 years of age). Additionally, gender, aetiology (trauma or no trauma), and SCI severity were analysed separately in the eligible population for each abovementioned hospital. Systematically, first every 10th person of the matrix who met the predetermined criteria was identified from the suitable group. Secondly, if the criteria were not met, the next most suitable person was selected. Additionally, the research group decided that every hospital area had to have at least one participant from each SCI severity group (although the incidence of SCI severity group was very low).

Participants in the FinSCI study were grouped by SCI severity based on the recommendations of The International Standards for Neurological Classification of SCI (ISNCSCI) [27], as well as in other publications [4,5,28]. The ISNCSCI is recommended to be used to determine the level and completeness of SCI by The American Spinal Injury Association Impairment Scale (AIS) [27], which grades the degree of impairment into five categories from A to E, where E indicates normal sensation and muscle function [29]. The severity of SCI is recommended to be reported in categories, where all patients with AIS grade D form one category. Patients with AIS grades A, B and C are recommended to be categorized into three groups by the level of injury: C1-C4, C5-C8 (cervical spine), and T1-S5 (thoracic and lumbar spine) [27]. SCI severity affects functioning since the higher the lesion is in the spinal cord the less function remains [28]. Additionally, persons with SCI and multiple injuries or a high level of lesions and AIS grades A, B, or C, have an increased length of stay in rehabilitation compared to those with lower SCI grades [30,31].

ST or KE contacted potential participants by phone. All who were asked to take part in interviews, and agreed to participate, were interviewed. The characteristics of the participants stratified by age and years since SCI are presented in Table 1 and the summary of characteristics in Table 2.

**Table 1.** Participants of the interviews in the Finnish spinal cord injury study (FinSCI), their general and lesion characteristics stratified by age and years since spinal cord injury *N* = 45.

Age	1-5 years since SCI	6-10 years since SCI	<11 years since SCI
20-30 years	**P5**, male, trauma, T1-S5 AIS A, B, C	**P1**, male, trauma, C1-4 AIS A, B, C	**P2**, male, non-trauma, C5-8 AIS A, B,C
	**P6**, female, trauma, AIS D	**P3**, female, trauma, T1-S5 AIS A, B, C	
		**P7**, male, trauma, AIS D	
31-45 years	**P8**, male, trauma, C1-4 AIS A, B, C	**P4**, male, non-trauma, T1-S5 AIS A, B, C	**P9** male, trauma, C1-C4 AIS A, B, C
	**P15,** male, trauma, AIS D	**P11**, male, trauma, C1-4 AIS A, B, C	**P10**, male, trauma, C1-C4 AIS A, B, C
	**P18**, male, trauma, AIS D	**P12**, male, trauma, T1-S5 AIS A, B, C	**P13**, male, trauma, T1-S5 AIS A, B,C
		**P16**, female, non-trauma, AIS D	**P14**, male, trauma, AIS D
		**P17**, female, trauma, AIS D	
46-60 years	**P23**, female, non-trauma, AIS D	**P25** male, non-trauma, AIS D	**P19**, male, trauma, C5-8 AIS A, B,C
	**P24**, male, non-trauma, AIS D		**P20**, male, trauma, C5-8 AIS A, B, C
	**P26**, male, trauma, AIS D		**P21**, female, trauma, T1-S5 AIS A, B, C
	**P28**, male, trauma, AIS D		**P22**, male, trauma, T1-S5 AIS A, B, C
			**P27**, male, trauma, AIS D
61-75 years	**P29**, female, non-trauma, AIS D	**P30**, male, trauma, AIS D	**P32**, female, non-trauma, AIS D
	**P33**, female, non-trauma, AIS D	**P31**, female, non -trauma, AIS D	
	**P34**, female, non-trauma, AIS D	**P35**, male, non-trauma, AIS D	
	**P36**, female, non-trauma, AIS D		
< 75 years	**P37**, male, trauma, C5-8 AIS A, B, C	**P41**, male, non-trauma, AIS D	**P42**, male, trauma, AIS D
	**P38**, male, trauma, AIS D	**P44**, female, trauma, AIS D	**P43**, female, non-trauma, AIS D
	**P39**, male, non-trauma, AIS D		
	**P40**, female, non- trauma, AIS D		
	**P45**, male, trauma, AIS D		

Note: SCI = spinal cord injury, Groups C1-4 AIS A, B, C ; C1-4 AIS A, B, C; T1-S5 AIS A, B, C and AIS D is the classification of severity of SCI based on the recommendations of The International Standards for Neurological Classification of SCI (ISNCSCI) [27].

**Table 2.** Summary of participant characteristics (*N* = 45).

		N
Gender	Females	15
	Males	30
Age in years	20–30	6
	31–45	12
	46–60	10
	71–75	8
	> 75	9
Time period since the injury in years	1–5	18
	6–10	14
	> 11	13
Etiology	trauma	28
	non-trauma	17
Severity of SCI	C1-C4 AIS A	5
	C5-C8 AIS A, B, C	4
	T1-S5 AIS A, B, C	7
	AIS D	29

Note: SCI = Spinal Cord Injury, Groups C1-4 AIS A, B, C; C5-8 AIS A, B, C; T1-S5 AIS A, B, C and AIS D is the classification of severity of SCI based on the recommendations of The International Standards for Neurological Classification of SCI (ISNCSCI) [27].

Tables 1 and 2 show that we were not able to completely implement the selection process as planned. The main reason for this was, that younger persons with SCI neither participated actively in the FinSCI study [4] nor were willing to take part in the interviews. There were 10 persons who declined to participate in interviews, and five of them were under the age of 30 years. For this reason, more persons were selected for the next age group (31–45 years of age).

### Data collection and analysis

Data were collected in semistructured interviews [32] between September and December 2019, primarily in the participants’ homes. Four participants were interviewed in a quiet workspace, which was organized by the interviewers at the participant’s request. Participants attended the interviews mainly by themselves, but for some, their partner or adult children also participated. Both interviewers were warmly welcomed, and the atmosphere during the interviews was open and natural. KE interviewed 22 participants and ST 23 participants. The similarity to interview questions was sought out through pre-preparations by interviewers and by using a semistructured interview guide (see Supplementary file A). Both interviewers, who are also primary researchers in this study, have a lot of knowledge of interviewing persons for research, and they also have a long work experience among persons with SCI. The interviewees were distributed between the ST and KE in such a manner that the respondents were not from the geographical area where interviewers worked to eliminate familiarity. The average duration of interviews was 1 h and 10 min (range: 45 min to 2 h and 54 min). The interviews were audiotaped, recorded, and transcribed verbatim by professional typists. In total, almost 53 h of interviews were accumulated, with 1500 pages of text generated. Before the actual analysis, ST and KE read and listened to the interviews and corrected the words that were unclear to the typists. After this, ST, KE, and SH made rules for the anonymization of the participants. ST or KE anonymized the interviews. SH read and accepted all the anonymized text data.

The deductive-inductive content analysis consisted of five steps, and an example of the analyses from phase two of the rehabilitation process, including citations from two participants, is presented in the Table 3. For the first step (deductive content analysis), ST used the Atlas.Ti version 9 program. ST assigned an identification number to each participant. As a theoretical framework for coding, ST used the six phases of rehabilitation process as described in Figure 1. KE checked 5 of the interviews that ST had coded, and no further citations for coding were found. After finalizing the coding with the Atlas.Ti program, ST exported the coded text data to Excel and added aetiology (TSCI or NTSCI) to the identification numbers since she assumed that aetiology had impact on the course of the rehabilitation process. ST created a matrix for each of the six rehabilitation phases, including the initial coding and the original citations from the text data. To enhance the credibility and confirmability [33] of this first step of the analysis, the primary researchers (ST and KE) discussed the analytical results until a consensus was reached and Step 1 was finished. After the deductive part of the analysis, ST started the inductive part. ST read original citations and initial coding several times, moving back and forth, to form subcategories for each citation (Step 2). These six matrices with original citations, initial codes, and subcategories served as the basis for the workshops, where the analysis was checked by the research group (Step 3). The members of the research group received the matrices 2 weeks prior to the workshop to familiarize themselves with the coded text data. In the workshop, the research group went through each citation and its analysis, discussed, and accepted or edited the subcategories suggested by ST. If there were differences in the interpretation of the citations among the research group members, the interviewer’s (ST or KE) interpretation was chosen as the name for the subcategory. The condition for categorization was that each category had to contain citations from at least two participants since the lack of multiple citations makes it difficult to conceptualize and identify a category [34]. Altogether, 6 workshops were held, and each lasted for 1 1/2 h. The phase 3 was felt to be particularly relevant in the analyses process since it defined subcategories for citations, and the use of a panel of co-researchers aimed to increase its content validation [24]. In the fourth step of the analysis, ST combined subcategories with similar meanings into categories, and as a final step (Step 5), the categories were combined into two main categories: either facilitators or barriers. Facilitators promoted progress in the rehabilitation process while barriers impeded it, making it difficult or even preventing it. The final two steps were approved by the research group. The study was carried out and reported according to the Consolidated Criteria for Reporting Qualitative Research [35].

**Table 3.** An example of the analysis process from deductive to inductive content analyses.

	Step 1	Step 2	Step 3	Step 4	Step 5
	Deductive content analyses	Inductive content analyses	Inductive content analyses	Inductive content analyses	Inductive content analyses
	Data review for content and coding / ST and checked by KE	Conceptualizing sub-categories / ST	Confirming / editing subcategories in workshops / Research Group	Combining subcategories into categories / ST and approved by the research group	Combining categories to main categories by marking them either as a facilitator (=F) or a barrier (=B) / ST and approved by the research group
Phase 2: Rehabilitation planning; rehabilitators own role, the role of experts, role of loved ones, universal design, costumized emplyment etc.*	*ST: Do you have a rehabilitation plan now? P2: Yes. I have twice a week rehabilitation. And now, they are home visits since my carpal tunnels were operated in the beginning of August. ST: Okay. Are they physiotherapy visits? P2: Yes, just physiotherapy. ST: I see, where was this rehabilitation plan made? P2: In Spinal Cord Injury Outpatient Clinic in the University Hospital. / P2 (10)*	Rehabilitation planning accomplished (in Spinal Cord Injury Outpatient Clinic)	Rehabilitation planning accomplished (in Spinal Cord Injury Outpatient Clinic)	Rehabilitation planning accomplished	F
	*KE: Well, why were you sent to (name of rehabilitation center), why didn’t you think of another option? P35: Well that’s all (professionals), we have like this Social and health care district -group, it was already being prepared in that way, it (rehabilitation center) is owned by hometown, so it was like automatically that they use it. KE:Yes P35: On paper it looks good. KE: Yeah, but was there any point discussed that you had an access to spinal cord-specific rehabilitation? P35: No…. I would have been ready to go even privately, …, I don’t think it was suitable for me, not at all, (it was) wrong with strength training, no, just useless, doing just little things, this six weeks, two months was wasted. / P35 (19)*	An unmotivated rehabilitation period impairs the progress of rehabilitation	Unmotivation does not describe the situation well enough. A new name for subcategory is: Inpatient rehabilitation did not meet the expectations of rehabilitee.	Deficiencies in rehabilitation planning	B

* As a theoretical background for the the deductive part of the anlyses we used the rehabilitation process by Autti-Rämö [21]. The rehabilitation process is divided into six phases and this example is from phase two.

## Results

**Figure 2.**
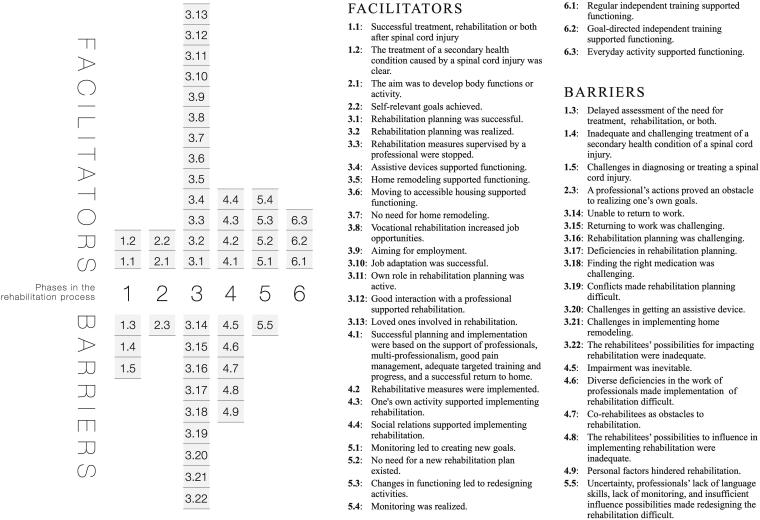
Summary of categories divided into six phases in the rehabilitation process. The phases of the rehabilitation process (Figure 1) were as follows: Phase 1: Applying and access to treatment or rehabilitation and approval of the need for rehabilitation; phase 2: Recognition of self-relevant goals and their concretization with professionals; phase 3: Rehabilitation planning; phase 4: Implementation of rehabilitation; phase 5: Monitoring achievement of goals and redesigning actions; and phase 6: Independent exercise in order to maintain the functioning.

Altogether, 887 citations from the rehabilitation process were analysed and categorized. The majority of the citations (650) were marked as facilitators, whereas 237 citations were marked as barriers. As a result of the analysis, 289 subcategories were conceptualized, which formed 47 categories. There was no clear difference in the saturation of categories between participants with traumatic or nontraumatic injuries. Most of the citations were related to rehabilitation planning (368) and implementation (275), whereas monitoring achievement of goals had the lowest number of citations (35). The subcategories, categories, and number of citations divided into six phases in the rehabilitation process are presented in Supplementary file B. Figure 2 summarizes all categories divided by phases in the rehabilitation process.

### Phase 1: Applying and access to treatment or rehabilitation and approval of the need for rehabilitation

The analysis of the first phase of the rehabilitation process revealed two categories that promoted rehabilitation: “Successful treatment, rehabilitation, or both after a spinal cord injury” and “The treatment of a secondary health condition followed by a spinal cord injury was clear.” The beginning of a rehabilitation process took place through acute and subacute care and rehabilitation in hospitals or rehabilitation centres, as well as in public health care. For example, one of the participants was pleased how his care and rehabilitation started and progressed immediately after the accident:

Well, it was stated right away that I am tetraplegic; nothing worked from the neck downward. They took me in an ambulance to the University Hospital, and I was in the intensive unit for about 1 1/2 weeks, and then I was in a ward for about two weeks before I got transferred to the rehabilitation center where I was for three months…. Mm, the memory is that I was very, very satisfied. I think everything was handled well. P7:1

The majority of participants who had secondary health conditions (SHCs) such as pain, spasticity, pressure ulcer, bladder problem, misalignment, due to their SCI, mentioned having good access and results concerning the treatment of these SHCs in their interviews. One participant described with positive humour the meeting between him and the staff in the hospital after receiving several treatments to different problems:

Baclofen pump was put in and removed when it became inflamed and then put in again. Then, I got a pressure ulcer in 2015, and I was operated on a dozen times before it had finally grown together…. Well, it has been… I do not remember which surgery it was, but in the University hospital, I went to the operating room and the staff had hardly seen me when they said, “are you here again?” I was like, yeah. P2:19

A delayed assessment for the need for rehabilitation was unpleasant for rehabilitees. Additionally, diagnosing or treating SCI was felt challenging, which guided rehabilitees to seek answers with the help of their friends or by themselves. The challenges included inappropriate treatment, self-payment for treatments, uncertainty about the rehabilitation place, additional injuries caused by SCI treatment, and difficulty in accessing special care due to SCI caused by a disease. This was exemplified by one participant who experienced delays several times, described as follows:

I had a job as a nurse at the health center, and then my legs started to go numb, so of course, I went to the company doctor. The company doctor then immediately said he would send me for a magnetic resonance imaging [MRI]… But then… it was getting worse and worse, and he [orthopedic doctor] said that it would probably have to be operated on. I called them twice in August… and then he came from his vacation to operate on me…. [After the operation and being home for a few weeks, the functioning got worse and a friend, who was physical therapist and had worked with persons with SCI, insisted that P33 calls the orthopedic doctor again]. So, I told him that I’ve gotten worse…Then, my friend shouted behind me that “she has paraparesis” … and he sent me for a new MRI the next day… they took an MRI of my whole back… and they found a tumor in the upper part of my back, which filled almost my whole spinal cord… I was sent right away for an operation in the University Hospital. P33:3-4

Difficulties in gaining access to treatments, and trusting the professionals, were described by one participant as follows:

Our health center is not working at all now, so you can never get an appointment there, and all the doctors have left because of social and health care messes. And the fact that if something were to happen to me, I would call the health center, but I must first find out [by myself] exactly what’s bothering me, and then I can call… P43:18

In addition to access to treatments, inadequate and challenging treatments for SHCs were seen as a barrier. The very same problems (pain, bladder function) that some participants perceived as being well treated were perceived as challenges by other participants, and all of them had TSCI. Autonomic dysreflexia and bowel function were difficult problems to solve, like the following example in which finding treatment for autonomic dysreflexia formed a barrier for a participant:

This autonomic dysreflexia is a horrible thing…it is a horrible, horrible thing, and in the beginning, there was not very much information about it. Nobody had heard about it, and my situation was so acute. In addition, there was not at all any kind of patience or understanding, and it was like they had never heard about it. You try to explain to the doctors and nurses, and then you cannot help yourself anyhow. P10:10

### Phase 2: Recognition of self-relevant goals and their concretization with professionals

As an aim for a treatment or rehabilitation participants reported maintaining or developing their body functions (joint mobility, muscle strength, balance, walking, weight management, fitness), receiving pain relief, increasing self-sufficiency, and maintaining functioning, as described in the follow statement:

[The goal is] to get rid of swelling… and then we try to keep the range of motion open, so that at least now it’s not going in worse direction… And then we take care of muscle strength so that it would stay good enough… And due to that I go independently to the gym… and swimming is always good. P9:25

Achieving self-relevant goals was a facilitator as well, and this happened during rehabilitation and with the help of peers or family members. In one example, one of the participants talked about setting and achieving his goals during the subacute rehabilitation period:

Well, to put it succinctly, I became independent. Like doing transfers, dressing, showering, all these daily things. I thought about it afterward, so in a way when I got out of there [subacute rehabilitation center], I was able to do everything the way I wanted. In addition, it was my goal there. That I said that I can’t be let out of here until things are in such a way that I can completely take care of everything myself. P13:10

Professionals’ actions were partly seen as a barrier in the realization of self-relevant goals: participants said that their goals were not considered, recognized, or achieved in their cooperation with professionals. Additionally, conflict in setting goals among professionals was reported as a hindrance to achieving self-relevant goals. A rehabilitee’s conflict between the high goals set by professionals, and her request for more help to learn to walk better and manage at home, is described as follows:

There were four or five male doctors who had the opinion that my walking with an aid went well; “you can move quite well, just go home” [with a tearful voice]. I’m such a gutsy person that if they say it to me once, and I have asked for help and I do not get it, I won’t ask a second time. So, I came home, and when I was being brought by ambulance-taxi in a laying position, the taxi chauffeur was about to drop me [laughs]. It was horrible; I texted my husband from there, lying in the taxi, to come and help me when the taxi came to the yard. I didn’t want to fall. P16:1

### Phase 3: Rehabilitation planning

The planning phase in the rehabilitation processes included 12 categories that facilitated rehabilitation and nine categories were barriers to rehabilitation. Some of the participants no longer had rehabilitation plans, so their rehabilitative activities related to SCI were finished. Several participants talked about how planning had taken place in health centres, university hospitals, occupational health care centres, or SCI outpatient clinics. Assistive devices and home modifications were perceived as facilitators for rehabilitation planning since having an appropriate living environment and useful aids made planning more practical. Collaboration and multidisciplinary teamwork were often mentioned as a part of a successful plan, described as follows:

There was good service there [subacute rehabilitation period in a university hospital]. I have nothing to say about it. [Interviewer ST: You said that there was always a big crowd of people sitting in the meetings?] I mean, everybody was there. [Nurse], physiotherapists, senior doctors, social workers, and everyone. That’s right, there was a big crowd. P15:12

In many interviews, a successful plan was followed by a good implementation of rehabilitation. The plan and implementation were interpreted as a success when a rehabilitee was pleased with a realization of both. This was recounted by one participant as follows:

Two years ago, I was there, [city name], at the SCI outpatient clinic. I was lucky, they gave me that three years like this [three-year rehabilitation plan] So I had physiotherapy 40 times and occupational therapy 30 times, and she [occupational therapist] comes to my home…It is nice that I don’t have to leave anywhere…And I understood that [in one year] I will go there [SCI outpatient clinic] again. P44:8

The educational changes and changes in the workplace related to work accommodations promoted rehabilitation, as well as the fact that the return to work was planned. Additionally, the active role of the rehabilitees and good cooperation among professionals facilitated rehabilitation planning. Working as an entrepreneur, having a part-time job, furthering their studies, and cooperating with an employee were facilitators of rehabilitation and promoted work accommodations, as explained in the following two examples where a vocational rehabilitation increased job opportunities (P1) and a job adaptation was successful (P34).

I believe that it is possible to get a job, and an organization for disabled persons is one where I would like to do all the practical periods and possibly work… Or get a job there… Because I feel that my strength is not to work in the field of youth work, but maybe on the administrative side; that’s why I’m at a university of applied sciences [now], so I can do those jobs someday. P1:27

Well, it progressed quickly. I went to our own doctor and said that the employer is agreeing to this [part-time work], and that I got the paper from there already…There must have been a smart doctor there as well reading the papers. [Interviewer KE: So you applied for a partial disability pension?] Yeah, yeah. That’s the 50%. And it came true. I work two days for one week and three days the second week. P34:7

The barriers in the planning phase of the rehabilitation process consisted of challenges and deficiencies. The reasons for these obstacles included ambiguities in the planning and organization of treatment, rehabilitation or both, a low amount of rehabilitation and guidance, conflicts among the rehabilitees and professionals or among professionals, environmental barriers and attitudes, and unfulfilled goals, among other things. An example of an ambiguity in planning was exemplified by one participant, whose head injury was overlooked, as follows:

I think a lot of things were overlooked in this accident. First, that person fell on my head, and I fell from standing up straight to the back of my head on the ground. Yes, I got a small scratch or wound on my head…Then I lost consciousness for a moment. My assessment is that I had a concussion because I got dizzy and vomited for a long time, but my condition was so serious that this was completely missed by everyone…And this thing with my memory, I’ve lost a bit of it, I don’t know what’s going on in my head if they don’t scan it. P28:4

Additionally, finding the right medication for pain and mental problems was perceived as challenging and time-consuming, thus hindering rehabilitation. Opportunities to influence rehabilitation planning were partly believed to be inadequate. Some of the participants talked about challenges in the realization of home remodelling and the acquisition of aids, which were believed to hinder the process. Deficiencies in the professionals’ work (insufficient language skills or knowledge about SCI) were experienced as a barrier, as stated by one participant who stopped his monitoring visits, since he felt that the professionals were more interested in promoting their own career than in his well-being:

I went there once [SCI outpatient clinic]. Twice actually. The first and the last time. The idea is probably very nice and good, but that… If you don’t get competence, permanent competence there. If a person comes into it, whose purpose is only to pursue their own interests, i.e., to seek effort from it to move forward, to profit from it, in the best case, just to auscultate, to get some final work done for themselves, or something else, then how on earth does it serve my cause, if it’s there on their own? P19:4

Challenges in returning to work were perceived as a hindrance, and reasons for these barriers included pain, depression, sleep problems, a lack of motivation, insufficient support, and environmental barriers, which were primarily related to the inability to move around in the workplace in a wheelchair or with the help of a walking aid. A few participants stated that it was not possible to return to work and that their work had not been adjusted because they had already been on sick leave before the SCI occurred. Sometimes barriers accumulated, such as the lack of motivation, pain, and depression, as stated by one participant as follows:

At the moment, at least, I don’t have the ability to work, and I don’t know if I ever will…But what I’m experiencing is that depression makes it sometimes so lacking in initiative that it feels like I can do that tomorrow, or not. I don’t start doing things like I used to. When the pain is at its worst, nothing really interests me in that moment. P12:14

### Phase 4: Implementation of rehabilitation

In the fourth phase of the rehabilitation process, more categories were discovered, which prevented the process (5) instead of facilitating the process (4). Facilitators, which were often described by participants, were included as a category: ‘Successful planning and implementation were based on the support of professionals, multiprofessionalism, good pain management, adequate targeted training and progress, and a successful return home.’ This means that progress in functioning, receiving pain relief, achieving goals, receiving support and guidance, multidisciplinary teamwork, and sufficient resources, among other things, were facilitating factors for rehabilitation. Being able to live at home after SCI motivated to rehabilitate. The implementation of rehabilitation took place in outpatient therapy sessions, health centres, inpatient rehabilitation centres, outpatient clinics and hospitals. This was described by one participant as follows:

First, I learned to walk. I was such in a bad condition that they took me with a wheelchair to a gym, and there was a really good instructor and a lot of different devices, and we started little by little…And then there were all these therapists of all kinds and so… It’s the best hospital I’ve ever been to. It was so incredible. [Interviewer ST: What kind of professionals did you meet? Like physiotherapists, sports instructors, or occupational therapists?] It was. Isn’t he the one who makes such block tests? Yes, it was. Then, a social worker. I think at some point, I had a speech therapist…There were nurses who helped with everything in the ward. And then there were those doctors. They visited me two or three times a day at best. P26: 5,6,8

Additionally, the participants’ own activities and social relationships supported the implementation of rehabilitation, described by a participant as follows:

I got such good acquaintances [a rehabilitation center during subacute rehabilitation] there. The spirit was quote famous, I remember it was a great place. You can say that it was such a communal place; that is to say, everyone was connected with everyone there, so it wasn’t so isolated…nobody was isolated, and no one became isolated. But all the time, we were together rehabilitating ourselves, so amazingly good friendships developed because of that. P27:5

As a main barrier in implementing rehabilitation measures during the process, the participants reported deficiencies in the work of professionals. The reasons were similar to those in the rehabilitation planning phase: a lack of knowledge, guidance, language skills, time, multidisciplinary teamwork, and suitable equipment or places and an inadequate amount of therapy received were seen as barriers. Professionals’ pejorative attitudes toward disabilities and the participants’ distrust toward professionals were also mentioned as preventing rehabilitation. Additionally, personal factors (young age, previous experiences with rehabilitation, anxiety about family members at home) and problems with co-rehabilitees were experienced as barriers to the process. Some participants had experienced several barriers simultaneously, like problems with co-rehabilitees and lack of inadequate amount of therapy, as described in the following example:

…it was quite a disaster. It was called a rehabilitation hospital. There were old people, and there were people who had a brain injury or circulatory disorder in their head or something else, who were really, really messed up… I made a patient injury report. Because, practically speaking, I rehabilitated myself there, because there were so many people in bad shape. People died there during the time I was there and… [Interviewer KE: For example, did a physiotherapist visit you?] I had a name, but there was the fact that they were moving to a new place, they were being trained. [speaks in a tearful voice] There really wasn’t anyone in sight for long periods during the day. Not even weekly. P36:2

The rehabilitees’ own abilities to influence the implementation of rehabilitation were partly seen as inadequate. This was described by one participant who was forced to change the physiotherapy provider due to competition legislation by Social Insurance Institution of Finland (Kela).

How the hell is this useful for me? Nothing at all. And I said to Kela that I can’t understand this… What the hell am I doing there and stretching my muscles when I have to manage at home? … in my previous physical therapy provider there were a lot of different kind of gym devices which I was able to train my leg and arm muscles, and on the other side they had this plinth where I could exercise a lot of other things. P32:5

### Phase 5: Monitoring achievement of goals and redesigning actions

There were four categories that promoted the rehabilitees and professionals to monitor achieving goals and redesign actions. Being monitored made it possible to the rehabilitee to plan new goals and notice the changes in the functioning. The benefits of planned, regular monitoring for rehabilitation were described by one participant as follows:

We do it in the way that she [physiotherapist] gives me [home exercise program], we have agreed so, and I go there every month and a half. It won’t help you at all if you don’t do them. We do this in cooperation. I have asked this, and she is satisfied; we make a program for me, which I carry out very obediently, because these are very important things to me. P35:9

There were also participants whose monitoring and rehabilitation planning were finished and participants who were still being monitored but had no new rehabilitation plan. Both categories were interpreted as facilitators since the rehabilitation process had progressed as planned, as described in the following quotation from a participant:

[Interviewer ST: And now every three years, you have those visits to the spinal cord injury clinic?] Yes, now is the first time that it is every three years. Earlier, it was every year… but the situation hasn’t changed anymore… Well, it has remained more or less the same, actually is not getting worse so quickly [as earlier] P42:5

There was one category that functioned as a barrier in monitoring and redesigning the rehabilitation process. A lack of language skills of and monitoring by professionals, as well as uncertainty and insufficient possibilities for exerting one’s influence, made it difficult to redesign the rehabilitation process. The need for monitoring and getting support to everyday life was expressed by a participant:

The monitoring, meaning the life after the spinal cord injury, that should be given more weight than the rehabilitation after the injury, which is a few months, maybe a year or two. Of course, there are in-patient rehabilitation available, but I think we need it more support for normal life. P4:19

### Phase 6: independent exercise in order to maintain functioning

The last phase of the rehabilitation process only included facilitative categories (3). Few participants performed goal-directed independent training and aimed for competitions or had other measured goals. Several participants talked about how regular independent training and everyday activities (handwork, outdoor activities, relaxing, etc.) supported their functioning, as recounted by one participant:

[Interviewer KE: You seem to have a standing frame [at home], how often do you use it?] Well, every day, every day. I also have an aid and a hand bike, so I do handcycling with it. I use it in the summer, now the season is over a bit. Then, I have one, it’s called a trainer. I bought it and can get it upstairs and pull it inside; it’s a bit more boring, but at least I get some exercise… well my injury is the kind of that it’s not getting away so it’s like just keeping up the condition, so I’m quite happy how I can do that. P5:11

## Discussion

The current study identified more facilitators (28) than barriers (19) in the rehabilitation process perceived by interviewed persons with SCI. Factors such as treatments or rehabilitation events, goals, multidisciplinary teamwork, support and monitoring in different situations (the need for aids, home or work remodelling, rehabilitation planning, etc.) and the rehabilitees’ own capabilities and activities facilitated the rehabilitation process. However, there were more barriers than facilitators during the initial phase of applying for and accessing treatment or rehabilitation, as well as during the implementation of rehabilitation. The barriers consisted of delays, challenges and deficiencies in the planning and implementation of treatment or rehabilitation, lack of skills and resources among rehabilitation professionals and different personal factors in rehabilitees.

Common for both facilitators and barriers were that they consisted of different communication and interaction situations, especially during the first five phases of the rehabilitation process. In other words, cooperation between different stakeholders when applying for rehabilitation, creating goals or plans, and implementing and monitoring them, was crucial for the progress of rehabilitation. There were many types of communication, which occurred especially between rehabilitees and professionals, in addition to with peers, family members, and friends. In a successful rehabilitation process, the facilitators followed each other, and several events promoted rehabilitation. Correspondingly, the barriers also followed each other and caused a vicious circle, in which the exact causes of the challenges were difficult to pinpoint. Thus, it can be concluded that the cornerstones of the rehabilitation process seem to be general in nature, although SCI itself can be seen as a primary reason for the need for rehabilitation.

Previous research has shown that communication and interaction have a central role in successful education, health care, and rehabilitation since patient-provider communication [19], communication gaps [8] and professionals’ lack of communication [14] were reported as barriers for persons with SCI. In studies where health care professionals were interviewed, these problems were noted. In the studies by Röthlisberger et al. [36] and Li et al. [37], communication among professionals was found to be a challenge; Delays in the communication of prescriptions between doctors and nurses led to inefficiencies [36], and poor multidisciplinary communication and collaboration were related to the quality of accelerated rehabilitation process [37]. In contrast, a study by Johnston et al. showed that good communication among the whole staff is a critical element in their work [38]. Additionally, in some studies where persons with SCI were interviewed, effective patient–provider communication [10] and enhanced communication between a patient and their care team [39] functioned as facilitators in their lives. Additionally, recent studies concerning SCI population [40–42], and their caregivers [43], stated that communication between rehabilitation professionals and persons with TSCI, or their caregivers, had a prominent role in the rehabilitation process supporting individuals with SCI with their adjustment and recovery to issues like body experience [40], intimacy [41] and sexual functioning [42]. These results support our study, which showed that good communication and interaction were crucial for the rehabilitation process.

Since there are few published qualitative data concerning facilitators and barriers in the rehabilitation process of the SCI population, it is interesting to briefly analyse the facilitators and barriers that have been found in the rehabilitation of patients with stroke, which is a leading cause of adult disability in the European Union. [44] In the integrative review of Forgea et al. [45] that concerned engagement in rehabilitation and in the systematic review and meta-analyses of Plant et al. [46] in which goal-setting during rehabilitation was analysed, communication was mentioned as a facilitator. The barriers to engagement in rehabilitation consisted mainly of the impacts of stroke, such as spasticity, physical impairments, and cognitive deficits. As facilitators, self-efficacy, therapeutic relationships (meaning communication, holistic care, support, and access to information) and motivating factors were mentioned [45]. As a conclusion to goal setting, it was stated that the current methods were not suitable, but effective communication between staff and patients and tailoring the goal-setting process to individual preferences, among other things, can facilitate rehabilitation [46]. Thus, we can state that the factors affecting rehabilitation are similar in many respects among persons with neurological conditions.

Another interesting result in our study was that even though the rehabilitation process contained many more facilitating factors than barriers, there were two phases in which there were more barriers. Difficulties during the first phase, applying and access to treatment or rehabilitation and approval of the need for rehabilitation, included delays in diagnosis, treatment or rehabilitation and challenges in finding suitable treatments for SHCs caused by SCI. There are several reasons for delays and challenges in treating SHCs. First, it is important to remember that SCI has an effect on a person’s body structure and functioning and can further negatively influence the person’s performance and capacity for activities and participation. Environmental factors might influence each component of functioning [47]. Second, Finland is undergoing a reform where wellbeing services counties will be responsible for health, social, and rescue services instead of municipalities and hospital districts. The reform has not been easy, as it has received criticism regarding the many challenges in its implementation [48]. Third, the number of persons diagnosed with SCI has increased in recent years, which has to do with the aging of the population, as well as increased awareness of symptoms and findings regarding SCI; even mildly injured patients are referred for treatment, rehabilitation and monitoring [4]. This fact has increased the need for care of persons with SCI. Fourth, based on our results, it can be stated that the diagnosis and treatment of SCI and its SHCs can be demanding. Professionals working with persons with SCI should have adequate education and expertise, which can be difficult to achieve due to the recent labour shortage in health care in Finland [49].

Diverse deficiencies in the work of professionals were the largest barriers to the implementation of rehabilitation; this was the fourth phase in the rehabilitation process, which had more barriers than facilitators. The professionals did not have enough expertise or time, or they did not guide or work in a multidisciplinary manner to promote the implementation of rehabilitation. Additionally, the rehabilitees had inadequate opportunities to influence the implementation of their rehabilitation, which was named a barrier because it relates to cooperation with professionals. We interpreted our results so that one of the root causes behind these challenging barriers was also the lack of better communication and interaction between professionals and rehabilitees. Based on a recent scoping review and thematic analysis of literature by Jesus et al. [26], professionals often perceive their care as being more person-centred than their patients do. The most important part in the process of rehabilitation is the person-professional dyad, which should include respectful, compassionate, and collaborative interaction [26].

The question remains: How can we promote good communication and interaction in the rehabilitation process? The rehabilitation process itself is not enough to achieve rehabilitation. It is important to understand that rehabilitation includes a learning phase for professionals and depends on a professional’s ability to create a good relationship with a rehabilitee. Such a relationship is confidential, committed, and goal-oriented and identifies the factors that have an effect on the rehabilitee’s situation and the achievement of their goals [21]. Professionals’ responsiveness to react to different situations, empathy and emotional support have an important role in rehabilitees’ participation in the rehabilitation process and can promote positive health outcomes [50]. To improve communication and interaction between rehabilitees and professionals, they all need time and understanding to learn from each other. It is very important that all those involved in the planning, implementation, and monitoring of rehabilitation have a positive, appreciative attitude toward one another and that they are motivated to work toward common goals, which are meaningful for rehabilitees [51].

## Strengths and limitations

As a strength of the study, we had a large amount of data, which were collected through interviews with 45 persons with SCI. The interviews and analyses were carefully planned and implemented, and all authors participated in the inductive steps. We found the research method suitable for this study. The theoretical reference framework we used helped to structure the analysis of the interviews, and the phenomena relevant to the rehabilitation process were identified in the data. The interviews took place face-to-face at the participants’ homes or at a place of their choice. The atmosphere during the interviews was confidential, and on many occasions, the participants spontaneously mentioned their experiences, successes, and hardships for discussion. Most of the authors had worked with persons with SCI for several years, and they were familiar with the issues they were analysing. One of the authors has an SCI, thereby bringing the viewpoint of a person with SCI to the analysis as well. Due to the factors mentioned above, we were able to reach the goal of saturation and redundancy across citations and categories, and we identified the most central categories to describe barriers and facilitators in the rehabilitation process. This study had a qualitative design, any generalization of the results should be carefully considered. As a limitation of the study, it should be mentioned that it was difficult to encourage younger participants to join the study, and the selection process was not realized exactly as planned, mainly for this reason. Additionally, the interviews with an independent researcher offered the rehabilitees an opportunity for a different kind of communication than usually with health professionals, which may have had an impact that perspectives related to interaction and communication were highlighted.

## Conclusions

We identified more facilitators than barriers in the rehabilitation process of persons with SCI. Successful treatment or rehabilitation planning and implementation, self-relevant goals and goal achievement, support, and monitoring changing situations in life, as well as good interaction and multiprofessionalism promoted rehabilitation as facilitative factors. The barriers were, on many occasions, the opposite of the facilitators. Difficulties in the planning and implementation of treatment or rehabilitation, the lack of different skills and resources of professionals, and different personal factors made the process difficult.

According to rehabilitees’ views, facilitators and barriers during the rehabilitation process consisted of different communication and interaction situations. The facilitative rehabilitation process included several successful communication and interaction situations between the rehabilitees and professionals. Since rehabilitation is a process in which the different phases are closely connected, even one barrier can significantly complicate the entire rehabilitation process and prevent rehabilitation from taking place. Solutions for the barriers experienced by persons with SCI should be sought in an interprofessional manner where a rehabilitee has an active and equal role as a primary stakeholder in the process.

## Supplementary Material

Supplemental Material

Supplemental Material

## Data Availability

An anonymized version of the data can be made available from the research leader (SH) upon reasonable request.
