# Supplementary material for: Facilitators and barriers in the rehabilitation process described by persons with spinal cord injury: a deductive-inductive analysis from the Finnish spinal cord injury study
Source: Ann Med. 2024 Jan 17;55(2):2303398. doi: 10.1080/07853890.2024.2303398 (PMC10795784; doi:10.1080/07853890.2024.2303398)
Supplement: Supplemental Material [file IANN_A_2303398_SM2143.pdf]

Additional file B. Tables 1–6: Rehabilitation process categories, subcategories, and number of citations

Rehabilitation phase 1: Applying and access to treatment or rehabilitation, and approval of the need for rehabilitation

Subcategory	Category	Number of citations	F=Facilitator B=Barrier
Access to treatment following an accident leading to a spinal cord injury was clear.<br>Access to treatment and subacute rehabilitation following an accident leading to a spinal cord injury was clear.<br>Access to treatment was clear when the cause for the treatment was found.<br>Access to public health care was achieved.<br>Diagnosis and access to treatment and subacute rehabilitation were clear after a spinal cord injury caused by a disease.<br>Access to treatment was clear after a recurrence of the disease that caused the spinal cord injury.<br>Long-term mobility impairment led to an assessment and acceptance of the need for rehabilitation.	1.1: Successful treatment, rehabilitation, or both after a spinal cord injury	44	F
Treatment for comorbidity causing a spinal cord injury was successful.<br>Treatment for the following comorbidities caused by a spinal cord injury was clear:<br>· pressure ulcer<br>· misalignment and pain<br>· urination<br>Guidance by a professional to a peer who helped find treatment.<br>Treatment of an injury resulting from a spinal cord injury was clear.	1.2: The treatment of a secondary health condition caused by a spinal cord injury was clear.	19	F
Diagnosis of the spinal cord injury caused by the disease was slow.<br>Delayed diagnosis and access to treatment after a spinal cord injury caused by a disease.<br>Diagnosis of a spinal cord injury caused by a disease and access to treatment is delayed; however, when a proper diagnosis is obtained, access to subacute rehabilitation was clear.<br>Access to treatment after a spinal cord injury caused by a disease was delayed because the cause of the symptoms was not found.<br>Delayed admission for subacute entry was due to interpretive differences in criteria.<br>Treatment after an accident leading to a spinal cord injury was delayed.<br>Applying for an estimate was slow due to incomplete research and one's unavailability.<br>Assessing the need for rehabilitation was delayed and required a struggle.<br>Delaying in diagnosing a spinal cord injury caused distrust in the rehabilitee.<br>Due to the remoteness and isolation of the accident's location—with illness being the primary cause—access to treatment was delayed.	1.3: Delayed assessment of the need for treatment, rehabilitation, or both.	20	B
Treatment of the following comorbidities caused by a spinal cord injury was inadequate and challenging:<br>· autonomic dysreflexia<br>· pain<br>· abdominal function<br>Treatment of comorbidity caused by a spinal cord injury was delayed.	1.4: Inadequate and challenging treatment of a secondary health condition of a spinal cord injury.	12	B

Diagnosis of a spinal cord injury caused by a disease was clear, but the treatment was demanding.<br>Access to treatment due to a spinal cord injury caused by a disease occurred at one's own expense.<br>Treatment of a spinal cord injury caused by a disease caused additional damage.<br>Inadequate treatment and assessment of the need for rehabilitation during an acute period caused distrust.<br>Uncertainty existed about the place of follow-up after surgery.<br>Access to special care was difficult due to the low spinal cord injury caused by the disease.<br>Assess to treatment failed.<br>Access to public health care did not work.<br>Obtaining rehabilitation and necessary services was unclear.	1.5: Challenges in diagnosing or treating a spinal cord injury.	10	B
**Rehabilitation phase 2: Recognition of self-relevant goals and their concretization with professionals**			
Subcategory	Category	Number of citations	F=Facilitator<br>B=Barrier
Rehabilitation's current goals were:<br>· Maintain body functions (joint mobility)<br>· Maintain an activity (fitness)<br>· Develop a fitness activity (walking, etc.)<br>· Develop a body function and an activity (balance and fitness)<br>· Develop a body function and an activity (fitness and joint mobility)<br>· Maintain body functions (reduce swelling, increse joint mobility, muscle strength)<br>· Maintain a body function and an activity (fitness, exercise)<br>· Maintain a body function and an activity (joint mobility, fitness)<br>· Maintain body functions (joint mobility, weight management, muscle work)<br>· Maintain functioning<br>· Relieve pain<br>· Increase self-sufficiency	2.1: The aim was to develop body functions or activity.	16	F
Self-relevant goals achieved:<br>· acquiring aid with the help of a peer due to treatment or rehabilitation<br>· learned new skills or became more independent during treatment or rehabilitation (despite challenges due to a subacute period)<br>· including one's family in the rehabilitation	2.2: Self-relevant goals achieved.	12	F
Professionals dis not consider current self-relevant rehabilitation goals.<br>A professional's actions proved an obstacle to realizing one's own goals.<br>Self-relevant goals were unachieved with a professional:<br>· in an acute hospital<br>· in rehabilitation<br>· in the acute hospital; however, the rehabilitee later accepted and learned to understand the professional's "prognosis"	2.3: A professional's actions proved an obstacle to realizing one's own goals.	21	B

The professional dis not recognize the goals that are relevant to the rehabilitee.			
Conflict existed among professionals in goal setting.			
**Rehabilitation phase 3: Rehabilitation planning**			
Subcategory	Category	Number of citations	F=Facilitator B=Barrier
Rehabilitation planning and implementation were successful:			
· in the health center and physiotherapy			
· in the hospital and physiotherapy			
· in the SCI-outpatient clinic and physiotherapy			
· in the hospital and rehabilitation center			
· in the regional hospital, physiotherapy, and pool therapy			
· continued in the home municipality, physiotherapy at home			
· at the SCI-outpatient clinic, health center, and physiotherapy, although the small numbers of appointments were annoying.			
Planning and implementing rehabilitation succeeded in cooperation guided by realistic goals.			
Rehabilitation planning and implementation were multi-professional and successful:			
· at the SCI-outpatient clinic and in physiotherapy			
· at the SCI-outpatient clinic and insurance company hospital			
· at the SCI-outpatient clinic and in physiotherapy and occupational therapy, but with a delay			
· during the subacute period			
· at the SCI-outpatient clinic, in physiotherapy, and during in-patient rehabilitation periods	3.1: Rehabilitation planning was successful.	52	F
· in the acute phase			
· in the postoperative period at the university hospital			
· in the hospital			
· at the SCI-outpatient clinic, in physiotherapy and psychotherapy			
· at the SCI-outpatient clinic, in physiotherapy, at in-patient rehabilitation periods, and possibility to psychotherapy			
Rehabilitation planning succeeded in multidisciplinary teamwork (SCI-outpatient clinic).			
Rehabilitation planning succeeded in collaboration:			
· with occupational health care			
· with operating hospital			
Rehabilitation planning succeeded:			
· at the SCI-outpatient clinic, although with a slight delay			
· when the physiotherapist encouraged it			
· at the SCI-outpatient clinic			
Successful access to rehabilitation services in cooperation (home municipality, The Social Insurance Institution of Finland, Kela).			

Rehabilitation planning was “just perfect” (SCI-outpatient clinic).			
Assistant or home care was involved in the planning, implementation, or both.			
The planning of rehabilitation was realized.<br>Rehabiliation planning was done,but some of the wishes of the rehabilitee were not realized.<br>Rehabilitation planning and monitoring carried out (SCI-outpatient clinic).	3:2 Rehabilitation planning was realized.	9	F
Rehabilitation measures supervised by a professional were stopped.	3.3: Rehabilitation measures supervised by a professional were stopped.	8	F
Assistive devices for functional support	3.4: Assistive devices supported functioning.	82	F
Home remodeling supported functioning.	3.5: Home remodeling supported functioning.	39	F
New home selected according to changed functioning.<br>Moving to accessible housing that supported functioning.	3.6: Moving to accessible housing supported functioning.	3	F
No need for home remodeling.	3.7: No need for home remodeling.	16	F
Vocational rehabilitation increased opportunities for work and accommodations.<br>Recognized in himself a need for vocational rehabilitation.	3.8: Vocational rehabilitation increased job opportunities.	8	F
Hope for a new job.<br>Return to work or accommodations was planned (studies at the moment).	3.9: Aiming for employment.	5	F
Part-time work and its accommodations made working possible.<br>Work accommodations succeeded:<br>· worked as an entrepreneur<br>· was self-employed<br>· studied a new field<br>Work accommodations succeeded:<br>· worked as an entrepreneur but recently stopped<br>· worked as a farmer but recently stopped<br>Successful work accommodations in cooperation with employers.	3.10: Job adaptation was successful.	23	F
Own role in rehabilitation planning was active.	3.11: Own role in rehabilitation planning was active.	5	F
Good interaction with a professional supported rehabilitation.<br>Encouragement from professionals to become independent was important.	3.12: Good interaction with a professional supported rehabilitation.	5	F
Loved ones plan on accompanying them to rehabilitation.<br>A loved one was involved in the planning, implementation, or both.	3.13: Loved ones were involved in the rehabilitation.	7	F
Unable to return to work or accommodate work for the following reasons:<br>· The participant experienced pain.<br>· The participant experienced pain and a lack of confidence.<br>· The participant "was unenthusiastic."<br>· For the participant, "time passed without work."<br>No accommodations to work (illness shortly before retirement).<br>No accommodations to work (on sick leave when spinal cord injury happened).<br>Work accommodations failed.	3.14: Unable to return to work.	12	B

Returning to work or work accommodations were challenging due tofor the following reasons:<br>· pain and depression<br>· pain and lack of confidence<br>· environmental barriers<br>Obtaining employment as a wheelchair user was challenging.<br>A return to work or work accommodations were planned, but inconsistent with the rehabilitee''s functioning and ability to work.<br>Professional guidance would have benefited the return to work / work accommodations.<br>Insufficient planning and support for vocational rehabilitation after coming home.<br>Continuing to work has been dictated by the own economy.	3.15: Returning to work was challenging.	23	B
Challenges in rehabilitation planning:<br>· in a subacute phase due to confusion concerning the payer<br>· in an outpatient spinal cord clinic because the doctor did not correctly understand the rehabilitee<br>· the plan was not implemented in the public sector<br>· among different actors (home municipality, city hospital, university hospital)<br>· in the subacute phase, the challenges were due to the rehabilitee's home situation and attitude towards other rehabilitees who were in poor condition<br>· in the spinal cord outpatient clinic and elsewhere due to the environmental barriers and attitudes a rehabilitee's experiences<br>· in the home municipality and city hospital, because rehabilitee was not understood by a doctor<br>· in the home municipality and city hospital, because getting appointments was difficult<br>· in the home municipality and with the Social Insurance Institution of Finland	3.16: Rehabilitation planning was challenging.	12	B
An unmotivated in-patient rehabilitation period impaired rehabilitation''s progress.<br>An in-patient rehabilitation period did not meet the rehabilitee's expectations.<br>Uncertainty existed in planning and organizing rehabilitation.<br>Rehabilitation planning was deficient.<br>No further rehabilitation was planned.<br>Deficiencies existed in rehabilitation planning by professionals.<br>Deficiencies existed in rehabilitation planning and inconsistencies existed among professionals and the rehabilitee during subacute periods (health center and in-patient rehabilitation center).<br>Loved ones did not received inadequate guidance.<br>Deficiencies in professionals planning and implementing rehabilitation: A visit to the spinal cord injury clinic was not immediately arranged after the injury.<br>Little guidance and rehabilitation were present.<br>Professionals had insufficient knowledge about spinal cord injury.<br>The rehabilitation plan did not lead to concrete action.<br>Deficiencies existed in planning acute care.	3.17: Deficiencies in rehabilitation planning.	14	B

Finding the right medication was time-consuming or challenging in the following health problems:<br>· mood<br>· problems with stomach function and pain<br>· pain	3.18: Finding the right medication was challenging.	7	B
Conflict in rehabilitation planning among professionals:<br>· The Social Insurance Institution of Finland did not accept the recommended amounts.<br>· Between the outpatient spinal cord clinic and insurance company<br>The Social Insurance Institution of Finland did not accept the recommended amounts, leading to decreased functioning.<br>Conflict existed in rehabilitation planning between the rehabilitee and a doctor.	3.19: Conflicts made rehabilitation planning difficult.	11	B
The assistive device succeeded only after new sufficient argumentations.<br>Obtaining an assistive device was troublesome.<br>Obtaining an assistive device was difficult since there was a conflict between professionals about the payer of an assistive device.	3.20: Challenges in getting an assistive device.	5	B
Home remodeling was completed with a delay.<br>Rehabilitee was dissatisfied with the extent of home remodeling.<br>Challenges presented in planning and implementing home remodeling.<br>All the rehabilitee's wishes were not considered due to:<br>· differing opinions among professionals<br>· uncertainty among professionals and the rehabilitee<br>· the lack of municipal technology<br>· the residential building's structure<br>· limited professional support<br>The rehabilitee had insufficient possibilities to influence the changes needed.<br>The home remodeling plan involved hopes for change.	3.21: Challenges in implementing home remodeling.	15	B
The rehabilitee's possibilities for impacting rehabilitation were inadequate.	3.22: The rehabilitee's possibilities for impacting rehabilitation were inadequate.	7	B

Rehabilitation phase 4: Implementation of the rehabilitation

Subcategory	Category	Number of citations	F=Facilitator<br>B=Barrier
Rehabilitation planning and implementation succeeded:<br>· in the occupational therapy<br>· during the subacute period<br>· during the acute period<br>· in the urology outpatient clinic<br>· at the health center<br>· in discussion help in the health center (5 x)<br>· in the outpatient physiotherapy (insurance company as a payer)<br>· Rehabilitation succeeded:			

· in the physiotherapy			
· in the city hospital, although there was uncertainty in the planning existed as home visits 5 x physiotherapy			
Rehabilitation planning and implementation succeeded (despite reduced physiotherapy appointments).			
Rehabilitation planning and implementation were successful.			
Set goals were reached.			
Things went well.			
Professionals had sufficient resources to carry out rehabilitation.			
Successful rehabilitation motivated oneself.			
Relieving pain and depressive symptoms helped implement rehabilitation.			
Successfull treatment of pain helped in rehabilitation.			
Pain treatment was essential in rehabilitation.			
Progress in the different areas of functioning motivated one to exercise.			
Increased self-sufficiency was due to rehabilitation.			
Good interaction with a professional supported rehabilitation.	4.1: Successful planning and implementation were based on the support of professionals, multi-professionalism, good pain management, adequate targeted training and progress, and a successful return to home.	115	F
Confidence in the skills of a professional helped with rehabilitation.			
Technical-assisted rehabilitation helped to develop different areas of functioning.			
Supported rehabilitation:			
· versatile physical training			
· walking training			
· practicing activities			
Instructions for self-practice supported rehabilitation.			
Being in rehabilitation was nice.			
Multidisciplinary work supported rehabilitation.			
Length and intensity of the program were adequate during the in-patient rehabilitation period.			
Skills accumulated due to rehabilitation.			
The return home was successful.			
Home remodeling was completed during the subacute period.			
The desired return home was an immediate success.			
The amount of rehabilitation was sufficient.			
The possibility of doing many more things promoted rehabilitation.			
A sufficient number of professionals brought support.			
Professionals had enough resources to carry out rehabilitation.			
The comfortable atmosphere was mentally refreshing.			
A professional supported independent training.			
Professionals supported implementing rehabilitation.			
Assistant promoteed implementing rehabilitation.			
Learning new skills in rehabilitation helped in everyday life.			
A driver"s license was received during the subacute period.			

The in-patient rehabilitation period disconneced from everyday life and this way supports rehabilitation.			
Rehabilitative measures were implemented (physiotherapy).<br>Implementing out-patient rehabilitation took time.<br>Rehabilitative measures planned and implemented:<br>· during a subacute period<br>· in a group training<br>· in a outpatient physiotherapy and occupational therapy<br>· at own expense in the private sector<br>The rehabilitation was carried out according to its current functional capacity.<br>Rehabilitative measures organized at the university hospital (physiotherapy).	4.2: Rehabilitative measures were implemented.	72	F
Own role is active in implementing rehabilitation .<br>The functions are maintained when one trains.	4.3: One’s own activity supported implementing rehabilitation.	6	F
Social contacts and peer support were important and motivating.<br>Social connections promoted rehabilitation.<br>Social relationships and peer support facilitated rehabilitation.<br>Peer support supported rehabilitation.	4.4: Social relationships supported implementing rehabilitation.	22	F
Self-sufficiency decreased over time despite rehabilitation measures.<br>Deteriorating functioning from time and excessive strain must be accepted.	4.5: Impairment was inevitable.	2	B
Rehabilitation was incomplete.<br>Deficiencies presented in implementing rehabilitation (no suitable place for in-patient rehabilitation exists).<br>Rehabilitation was incomplete:<br>· subacute period in the regional hospital<br>· more occupational therapy and practice of fine motor skills practice<br>· the number of therapies was insufficient<br>Lack of mental support weakened implementing rehabilitation.<br>Deficiencies in implementing rehabilitation by professionals:<br>· in the rehabilitation center of the healthcare district’s rehabilitation center<br>· in the subacute period after illness<br>Insufficient support or guidance of professionals (rehabilitation of intestinal function in the subacute phase).<br>Deficiencies in the work of professionals to consider all challenges of functioning.<br>Insufficient support and guidance from professionals (rehabilitation of bladder and bowel function).<br>Implementing rehabilitation was partly incomplete since there is no progress in the subacute period.<br>Rehabilitation was partially deficient because the:<br>· the number of therapies is too few<br>· at a private service provider, unsuitability of research equipment	4.6: Diverse deficiencies in the work of professionals made implementing rehabilitation difficult.	42	B

Planned in-patient rehabilitation periods ended when the payer estimated they were no longer beneficial.			
The hurry and lack of language skills of the healthcare professionals made rehabilitation difficult (Health District Rehabilitation Center).			
Rehabilitation was (very) deficient at the:			
· at the subacute period in the rehabilitation center			
· at the acute period at the hospital			
Multidisciplinary teamwork was unrealized.			
Feelings of insecurity were often present during rehabilitation.			
The rehabilitee's goals were not considered.			
The professionals had insufficient time.			
Distrust towards professionals existed.			
Too little time and insufficient rehabilitation did not promote rehabilitation.			
Early return to home without rehabilitative measures made rehabilitation difficult.			
Lack of mental support weakened the implementing of rehabilitation implementation.			
Guidance on rehabilitation measures was not provided.			
Professionals had a pejorative attitude towards disability.			
Rehabilitation was partly incomplete due to restless roommates.	4.7: Co-rehabilitees as obstacles to rehabilitation.	4	B
Excessive alcohol consumption by others disturbed rehabilitation.			
Restless roommates were scary.			
Peers do not support rehabilitation.			
The rehabilitee's rehabilitation opportunities were inadequate.	4.8: The rehabilitee's possibilities to influence in implementing rehabilitation were inadequate.	9	B
Previous bad experiences made focusing on rehabilitation periods difficult.	4.9: Personal factors hindered rehabilitation.	3	B
Anxiety about family members at home made rehabilitation difficult.			
Restrictions on participation caused by nosocomial infection reduced the chances of recovery.			
Rehabilitation in adolescence must be assessed individually.			
**Rehabilitation phase 5: Monitoring the achievement of goals and redesigning actions**			
Subcategory	Category	Number of citations	F=Facilitator B=Barrier
The success of a holiday at home during an in-patient period accelerated the homecoming.	5.1: Monitoring led to creating new goals.	15	F
The monitoring made appreciating one's achievements visible.			
Results beget satisfaction.			
Monitoring goals and redesigning activities were realized:			
· in the physiotherapy but delayed			
· at the spinal cord outpatient clinic			
No need for a new rehabilitation plan existed (on the rehabilitee's behalf).	5.2: No need for a new rehabilitation plan existed.	3	F
No goals are monitored, and no rehabilitation is redesigned (spinal cord outpatient clinic); however, access to health services (at the health center) is clear when needed.			

Changes in functioning led to redesigning activities, which has been difficult.<br>Changes in functioning led to redesigning activities with the help of assistants.	5.3: Changes in functioning led to redesigning activities.	2	F
Monitoring is realized:<br>· although no new goals or rehabilitation are planned<br>· at the neurosurgeological unit	5.4: Monitoring is realized.	7	F
Inadequate monitoring of rehabilitation caused uncertainty.<br>The rehabilitee had insufficient influence (for redesigning the rehabilitation).<br>Uncertainty existed about continuing rehabilitation (rehabilitative activities causes anger).<br>The lack of a professional''s language skills made monitoring and setting new goals difficult.<br>Regular monitoring and a comprehensive assessment are needed to find the necessary support for everyday life.	5.5: Uncertainty, professionals' lack of language skills, lack of monitoring, and insufficient influence possibilities made redesigning the rehabilitation difficult.	8	B
**Rehabilitation phase 6: Independent exercise in order to maintain the functioning**			
Subcategory	Category	Number of citations	F=Facilitator<br>B=Barrier
Regular independent training supported functioning.	6.1: Regular independent training supported functioning.	30	F
Goal-directed independent training supported functioning.	6.2: Goal-directed independent training supported functioning.	3	F
Everyday activity supported functioning by:<br>maintaining fitness and mental well-being<br>· maintaining fitness<br>· maintaining left-handed functions<br>· maintaining social relationships and mental well-being (watching hockey)<br>· engaging in reading and breathing exercises<br>· maintaining fitness by swimming in a fitness group and jogging<br>· maintaining fitness and manual skills by jogging and woodworking<br>· keeping the pain away<br>· maintaining fitness by participating in outdoor activities<br>· facilitating conscious awareness of keeping the body fit<br>· relaxing (using warm water)<br>Everyday activity supported functioning, although the number of assistance hours was insufficient to accomplish everything desired.	6.3: Everyday activity supported functioning.	22	F
		887	
