# Supplementary material for: Facilitators and barriers in the rehabilitation process described by persons with spinal cord injury: a deductive-inductive analysis from the Finnish spinal cord injury study
Source: Ann Med. 2024 Jan 17;55(2):2303398. doi: 10.1080/07853890.2024.2303398 (PMC10795784; doi:10.1080/07853890.2024.2303398)
Supplement: Supplemental Material [file IANN_A_2303398_SM2090.pdf]

Additional file A. Interview guide

Hello, thank you for letting me interview you. This interview is part of the FinSCI study investigating the health, functioning and accessibility challenges of people with spinal cord injuries. Here I have attached the statement of the ethics committee regarding the permission to conduct this research.

Also, I have a bulletin here for you to read first. The bulletin remains with you and it also contains our contact information. The bulletin states e.g. how the information obtained from the interview is stored and processed. All information collected during the interview is confidential. All information related to the person's identity is excluded from the study.

We have a maximum of one and a half hours for the interview. If you want to end the interview at some point, that is allowed. We have chosen 6 different themes for the interview, they are:

• *Rehabilitation and its different stages*
• *Returning home, housing, and type of housing*
• *Functioning and its importance in different areas*
• *Employment situation and paths to employment*
• *Accessibility and related factors*
• *Quality of life and well-being*

Which of these would you like to start with?

• Rehabilitation and its different stages
• Acute stage: The hospital period immediately after the injury.
o Where and when did you get injured?
o Where were you initially rehabilitated?
o Do you remember anything special during the rehabilitation that you would like to share

• Subacute stage: Acute rehabilitation period after the injury
o Where were you in rehabilitation?
o How long did the rehabilitation take?
o What did you learn in rehabilitation?
o Did you miss any skills that you felt you needed?
o Who rehabilitated you?
o Do you remember anything special during the rehabilitation that you would like to share

• Now
o Do you have a rehabilitation plan?
o Are you currently undergoing rehabilitation?
o Where and how often?
o Who pays your rehabilitation?
o What are you aiming for with rehabilitation?
o Are you satisfied?
    -if you are, what makes you satisfied
    - If not, what makes you dissatisfied
o Is there anything else related to rehabilitation that you would like to share

• Returning home, housing and type of housing
o Where did you move after the injury?
o Who did you live with?
o What was your home like (remodeling, etc.)?
o Have you moved often since then?
- if you have, why?

o What kinds of things are you paying attention to in your home now?
o Who do you live with now?
o Is your home right for you?
o What kind of changes would you like to have in your home?
o Is there anything else related to home and living that you would like to share

• Functioning and its importance in different areas
o What does your typical day like?
o What activities do you do by yourself daily?
o What activities do you need help with?
o What kind of aids do you use?
o Who helps you?
o Do you get enough help?
o Are you satisfied with what you can do independently?
- If not, what would you like to do and how would you do it?
- If you are satisfied with the way you function, what is important for you to be able to do by yourself and why?

o Is there anything else related to your functioning that you would like to share

• Employment situation and paths to employment
o Were you working before the injury, if so where?
o What kind of education do you have for your profession?
- Have you received additional training or in a new field after your injury?

o Have you worked since the injury, if so, where?
o If you can no longer work, how has it felt to give up work?
- What reasons have led to the fact that you have not been able to do work?

o If you are still working, what does working mean to you?
o What kinds of things have contributed to your employment?
o Are there challenges at work that are related to your injury?
o Is there anything else related to working that you would like to share
o Your wishes regarding employment or work-related life situation

• Accessibility and related factors
Accessibility makes it possible for people to live in their own home and participate smoothly in work, hobbies, culture, and studies. Accessibility also means, among other things, the accessibility of services, the usability of tools, the comprehensibility of information and the possibility to participate in decision-making about oneself. Accessibility is equality and part of sustainable development. Accessibility is not just about the accessibility of movement. It also considers issues related to vision, hearing, communication, and electronic communication, for example.

o What kind of accessibility-related challenges have you faced?

- Physical barriers, regulations and laws, people's attitudes,

o Is your injury the main reason for the obstacles?
o How have you solved challenges related to disability?
o Have you missed doing/accomplishing something because of a disability?
o What kind of accessibility solutions/methods do you use in your everyday life?

- For example, do you always go to a certain store because of its accessibility?
- Do you prefer a service provider because he has a "good attitude"? And so on.

• Quality of life and well-being

o How do you feel about your quality of life now?
o What do you think a good quality of life is like?
o Has your quality of life changed since you were injured?

- If so, how?

o What things are important to you?
o Are you able to influence on things that are most important to you?
o How do you see your future?

Now we are almost ready with this interview. I repeat that your participation in this study is voluntary and confidential. And you can also cancel your participation in the study at any time without consequences. All information that could lead to the identification of a person is left out of the research material. If you have any questions, you can contact me, and the contact information can be found in the bulletin. I will contact you later regarding the processing of the entire interview material, i.e. I will present the preliminary results, which you will have the opportunity to comment on. Thank you!
